# Cyberbullying Victimization and Adolescent Depression: The Mediating Role of Psychological Security and the Moderating Role of Growth Mindset

**DOI:** 10.3390/ijerph17124368

**Published:** 2020-06-18

**Authors:** Gengfeng Niu, Jing He, Shanyan Lin, Xiaojun Sun, Claudio Longobardi

**Affiliations:** 1 School of Psychology, Central China Normal University, Wuhan 430079, China; niugfpsy@mail.ccnu.edu.cn (G.N.); psyhejing@163.com (J.H.); 2 Key Laboratory of Adolescent Cyberpsychology and Behavior (CCNU), Ministry of Education, Wuhan 430079, China; 3 Center for Research on Internet Literacy and Behavior, Central China Normal University, Wuhan 430079, China; 4 Department of Psychology, University of Turin, 10124 Torino, Italy; claudio.longobardi@unito.it

**Keywords:** cyberbullying victimization, depression, psychological security, growth mindset, adolescents

## Abstract

The objective of this study was to examine the mechanisms (the mediating role of psychological security and the moderating role of growth mindset) underlying the association between cyberbullying victimization and depression among adolescents. A sample of 755 adolescents (M_age_ = 13.35 ± 1.02; 373 boys) was recruited from two junior high schools, and the participants were asked to voluntarily complete a set of measures, including the cyberbullying victimization subscale in the Chinese version of the Cyberbullying Inventory, the Chinese version of the Security Questionnaire, the Chinese version of the Center for Epidemiologic Studies Depression Scale, and the Growth Mindset Inventory. The results indicated that: (1) cyberbullying victimization was positively associated with depression through the mediating effect of psychological security and (2) both the direct association between cyberbullying victimization and depression and the indirect association through the mediating effect of psychological security were moderated by growth mindset. Specifically, growth mindset could significantly alleviate the adverse effects of cyberbullying victimization on psychological security and on depression. These findings not only shed light on the mechanisms linking cyberbullying victimization to depression among adolescents, but also provide an empirical basis for formulating prevention and/or intervention programs aimed at reducing depression levels and the negative influences of cyberbullying victimization among adolescents.

## 1. Introduction

In the current information age, many negative phenomena (such as Internet addiction and cyberbullying) have emerged with the increasing popularity of the Internet. Cyberbullying refers to intentional, repetitive, hostile, or aggressive behaviors to inflict harm or discomfort on others through electronic or digital media, such as e-mails, instant messaging, and social networking sites [1,2]. Recently, it has become a serious social problem and public health concern around the world due to the seriously detrimental influences of cyberbullying victimization (i.e., being victimized online) on individual social adaptation and well-being [3,4,5]. At the same time, depression is a common psychological problem with high prevalence [6], which has been found to be a great risk factor for individual health and well-being. Depression not only causes acute emotional pain, interpersonal relationship dissatisfaction, and social dysfunction, but also is closely associated with suicidal intentions and behaviors [7,8]. Against this background, exploring the antecedent factors of depression has always been a focus of relevant studies, and more attention has been paid to the relationships between Internet-related factors and depression [8,9]. Especially, the ecological techno-subsystem theory (which expands the ecological systems theory in Development Psychology) points out that modern information technology (especially the Internet) has become a component of the ecological system influencing individual development and adaptation [10].

Social relationship plays an important role in influencing depression [11,12,13]. As a negative experience in social relation, bullying victimization was found to be closely associated with depression, and both cross-sectional and longitudinal research indicated that individuals who had experienced bullying victimization or were being bullied, were more vulnerable to depression [14,15,16]. For cyberbullying, as a specific form and extension of bullying in online space, though it is different from the bullying in real life (or traditional bullying), being bullied by others online is also a negative and stressful experience in social interaction [5]. In particular, because of the unique features of cyberbullying (such as the anonymity of the bully, the publicity of the bullying behaviors, and the widespread information used to bully), being bullied online is even more stressful than being bullied traditionally, and thus cyberbullying victimization may induce more serious consequences [3,4]. Empirical research also found that cyberbullying victimization was associated with some psychological problems (e.g., depression) [2,3,17].

Adolescence is a vulnerable period in terms of depression. The relevant studies also found a significant increase in the prevalence rate of depression in adolescence [18]. At the same time, due to the high frequency and intensity of adolescents’ online activities, cyberbullying victimization is relatively more pervasive among adolescents [3,17]. Thus, this study aimed to examine the association between cyberbullying victimization and depression among adolescents, and it was also hypothesized that cyberbullying victimization was positively associated with depression (H1). In addition, with the deepening of research, more and more attention is being paid to the intervention of cyberbullying, however current intervention mainly focus on cyberbullying neglecting the victims [19,20]; at the same time, the mechanisms underlying the influences of cyberbullying victimization on individual adaptation is also one of the research focus, which is beneficial for the development and design of intervention programs aimed at reducing the negative influences of cyberbullying victimization and beneficial for the development of prevention and intervention programs for depression associated with cyberbullying victimization. Thus, this study further aimed to examine the association between cyberbullying victimization and depression among adolescents, as well as the underlying mechanisms (i.e., how cyberbullying victimization was linked to depression and the potential individual differences in this association). 

### 1.1. The Mediating Rrole of Psychological Security

Psychological security refers to the feelings of safety and belongingness, as well as a sense of control over the social environment and confidence in being free from fear [21]. As one of the most important psychological needs, it is the basis of individual well-being and mental health [22,23]. Individuals with poor psychological security may feel rejected and isolated, and further perceive the outer world and other people as threatening, untrustworthy, and uncontrollable [24]. These negative perceptions and feelings may induce them to form a gloomy view of the future and life and can further lead to maladaptive outcomes, such as problem behaviors (e.g., Internet addiction), anxiety, and poor academic performance [11,25,26].

As for depression, though the current studies have not directly examined its relationship with psychological security, relevant studies can provide indirect evidence. First, studies showed that the negative perceptions of social relationships (e.g., perceived social isolation and poor interpersonal trust) and the social environment (e.g., a sense of control) were significantly associated with depression [9,27]; additionally, all these negative social perception factors were closely related to psychological security [24,26]. Second, researchers indicated that low level of psychological security was a main risk factor for suicidal ideation, suicide attempts, and suicidality [28]. Depression is also closely related to suicide [8]. Thus, psychological security may also be significantly associated with depression.

At the same time, psychological security is directly affected by social environmental factors. Numerous studies have indicated that negative interpersonal contexts and relationships will cause individuals to hold negative beliefs about the world (e.g., the world is unpredictable and uncontrollable) and self (e.g., they are isolated), thereby greatly impairing their psychological security [22,28,29]. Regarding bullying, relevant empirical studies demonstrated that bullying victimization was negatively associated with psychological security and that adolescents victimized by peers tended to report lower levels of psychological insecurity [11,28]. As discussed above, cyberbullying victimization is also stressful and can also induce deleterious consequences [3,4,17], especially adolescents who are victimized online tend to feel powerless in terms of protecting themselves, and even helpless regarding the future and the environment [17,29]. Thus, cyberbullying victimization may be negatively associated with psychological security.

Furthermore, psychological security may also serve as a key mediator underlying the association between negative interpersonal experience and adaptation. In particular, emotional security theory proposes that negative interpersonal contexts (e.g., hostility and aggression) threaten individuals’ psychological security, which in turn leads to various maladaptive outcomes [25,30]. Empirical studies on bullying have also supported this theory, for example, Peng et al. [28] found that bullying victimization could influence adolescent suicidal ideation through the mediating role of psychological security. Thus, it was further hypothesized that psychological security would mediate the relationship between cyberbullying victimization and adolescent depression (H2).

### 1.2. The Moderating Role of Growth Mindset

In addition, this study also aimed to investigate the individual differences in these associations. Especially, the diathesis–stress model of depression points out that the dynamics of the individual–context interactions are the key mechanisms that contribute to developing depression [6,31]. It is also of great significance to explore the positive individual factors buffering the deleterious effects of cyberbullying victimization on depression.

Mindset (beliefs about the malleability of personality attributes) is one of the key factors determining individual responses to challenges, stress, and adversity [32], and it is one focus of the current research in related fields. In particular, growth mindset, as a specific form of mindset, refers to the belief that personal attributes (e.g., personality, strength, and skill) are changeable rather than immutable [33]; and it has been found to be a positive predictor for various aspects of individual adaptation and well-being [34,35,36]. At the same time, growth mindset enables individuals to adopt adaptive strategies, such as cognitive reappraisal, to cope with negative situations and environments; therefore, individuals with growth mindset tend to be more adaptive when facing negative experiences [33,37]. Empirical studies also found that growth mindset could moderate the relation between negative experiences (e.g., stressful life events and social exclusion) and mental adaptations (e.g., psychological distress, anxiety, and aggression), and this association is weaker among growth-minded individuals [37,38]. Besides, the stress-buffering model also indicates that positive individual and environmental factors could alleviate the negative impacts of stressful contexts on individual adaptation. The moderating or buffering role of positive personal traits (e.g., resilience and mindfulness) in the association between negative interpersonal experiences (e.g., being social excluded and bullied) and individuals’ feelings (e.g., helplessness) and adaptation (e.g., depression) has been well examined in previous studies [12,17,28,39]. Considering this evidence, it was further hypothesized that the direct association between cyberbullying victimization and depression, as well as the mediating effect of psychological security, could be moderated by growth mindset (H3).

To sum up, based on relevant studies, a moderated mediating model was constructed to examine the mechanism underlying the association between cyberbullying victimization and depression the mediating role of psychological security and the moderating role of growth mindset.

## 2. Materials and Methods 

### 2.1. Participants

Participants were recruited from two junior high schools (from grade seven to grade nine) in a city of central China with the approval of the school authority and the Academic Committee for Scientific Research at the first author’s university. Convenience sampling method was adopted, in particular, two classes in each grade were randomly selected from the two schools, and the students in the selected classes were invited to participate in this study. In the end, a total of 755 adolescents (373 boys and 382 girls) aged between 12 and 15 (Mage = 13.35 ± 1.02) participated in this study voluntarily. 

### 2.2. Measurement

#### 2.2.1. Cyberbullying Victimization

The cyberbullying victimization subscale in the Chinese version of the Cyberbullying Inventory [5] was adopted to assess participants’ cyberbullying victimization. The subscale consists of 18 items, each of which describes a specific cyberbullying victimization behavior (e.g., “Someone spread rumors about me online”), and participants were asked to assess how often they had encountered the cyberbullying victimization activities in the past year on a 4-point Likert scale ranging from 1 (never) to 4 (more than five times). Cronbach’s alpha for the subscale in the current study was 0.85.

#### 2.2.2. Psychological Security

The Chinese version of the Security Questionnaire [11,24] was adopted to measure participants’ psychological security. This questionnaire consists of 16 items, which can be divided into two dimensions—interpersonal security (e.g., “I never dared to say what I thought”, reverse coded) and certainty in control (e.g., “I always worry that my life will be a mess”, reverse coded). Participants were asked to respond on a 5-point Likert scale ranging from 1 (extremely non-compliant) to 5 (very compliant). Cronbach’s alpha for the subscale in the current study was 0.86.

#### 2.2.3. Growth Mindset

The Growth Mindset Inventory [33] was adopted to measure adolescents’ thoughts regarding growth mindset. It consists of eight items (e.g., “Though I may make mistakes, I like doing tasks from which I can learn new things”). Participants were asked to respond on a 5-point Likert scale ranging from 1 (strongly disagree) to 5 (strongly agree). This inventory has been used among Chinese adolescents and has good reliability and validity [36]. In this study, a confirmatory factor analysis revealed an acceptable fit: χ^2^/df = 4.55, RMSEA = 0.07, AGFI = 0.92, GFI = 0.93, IFI = 0.93, CFI = 0.93; Cronbach’s alpha for the subscale in the current study was 0.81.

#### 2.2.4. Depression

The Chinese version of the 20-item Center for Epidemiologic Studies Depression Scale (CES-D) [40] was adopted in this study. Respondents were asked to assess how often they had been bothered by each item over the last week on a 4-point Likert-type scale. In our study, Cronbach’s alpha for the scale was 0.88.

### 2.3. Procedure

This is a cross-sectional correlational survey, which has been carried out during the first semester of 2019–2020 academic year. A paper–pencil questionnaire was filled out during class hours, before which a standardized introduction to this study was presented. It took 10 to 20 minutes to complete this questionnaire, and students were compensated with a small gift (approximately 0.5 US dollars) after the study.

### 2.4. Ethics Approval

This study was approved by the Ethical Committee for Scientific Research at the researchers’ affiliated institution. The ethical values required in research with human beings, the fundamental principles included in the Helsinki Declaration (e.g., informed consent, protection of personal data, and guarantees of confidentiality), as well as the regulations of the education management department were followed. At the same time, all the participants and their parents were informed of the principles of the study, and parental consent for the children’s participation in the study was also obtained.

### 2.5. Statistical Analysis

All the statistical analyses were conducted with the SPSS 23.0 (IBM Corp, Armonk, NY, USA) and the Mplus 8.0 (Muthén & Muthén, Los Angeles, CA, USA) software packages. First, confirmatory factor analysis for a single factor was conducted to test the common method bias because all the data were collected via questionnaires. A normality test was also conducted to examine whether the research data were normally distributed. Then, the descriptive statistics were computed for the main study variables, and Pearson’s correlation analysis was also conducted to examine the relationships among the variables. Third, the PROCESS macro [41], which was developed and widely used to test complex models with moderating and mediating effects, was adopted to test the hypothesized moderated mediation model with 5000 bias-corrected bootstrapped samples from the original data. These bootstrapped samples were used to estimate the 95% confidence interval (CI), and the effect is considered significant if the 95% confidence interval values do not include zero. Specifically, Model 4 was conducted to test the mediating model with psychological security as the mediator. Afterward, Model 8 was conducted to test the integrated model with psychological security as the mediator and growth mindset as the moderator.

## 3. Results

### 3.1. Test for Common Method Bias and Normality

According to relevant suggestions, confirmatory factor analysis was conducted to test the hypothesis that a single factor can account for all the variance in the study data [42]. The results revealed a poor model fit (χ^2^/df = 15.76, RMSEA = 0.31, TLI = 0.52, CFI = 0.57), indicating that there was no serious bias in the estimation of the relationship between constructs. This meant that the significant influence of the common method bias on the results could be excluded and the reliability and accuracy of the research results could be ensured.

Then, the Kolmogorov-Smirnov test was adopted to check the normality of the research data. In the current study, all the skewness values were below 2.0, and all the kurtosis values were below 7.0, which indicated that the data were normally distributed [43].

### 3.2. Descriptive Statistics and Correlations between Main Study Variables

The correlation analysis showed that all the main research variables were significantly correlated with each other.

As shown in Table 1, cyberbullying victimization was positively correlated with depression (*r* = 0.36), while negatively correlated with psychological security (*r* = −0.39) and growth mindset (*r* = −0.22); psychological security (*r* = −0.48) and growth mindset (*r* = −0.29) were negatively correlated with depression; and psychological security was positively correlated with growth mindset (*r* = 0.30).

### 3.3. Testing the Hypothesized Model

As previous studies indicated that gender and age were closely associated with depression [6,8], they were included in the analysis as control variables. First, the mediating model analysis showed that, after controlling for age and gender, cyberbullying victimization was negatively associated with psychological security (*β* = −0.32, *p* < 0.001), while positively associated with depression (*β* = 0.28, *p* < 0.001), and psychological security was also negatively associated with depression (*β* = −0.41, *p* < 0.001); additionally, the direct association between cyberbullying victimization and depression was also significant (*β* = 0.16, *p* < 0.001), indicating that psychological security partially mediated the relationship between cyberbullying victimization and depression (indirect effect = 0.12, 95% CI = 0.06 −0.21). These results supported the hypothesis 1 and 2.

Then, Model 8 of Hayes’ SPSS macro PROCESS [41] was conducted, and the results are presented in Table 2 and Table 3. The results of the regression models (see Table 2) showed that, after controlling for age and gender, cyberbullying victimization was negatively associated with psychological security (*β* = −0.26, *p* < 0.001), and psychological security was negatively associated with depression (*β* = −0.36, *p* < 0.001), which further verified the mediating role of psychological security. These findings also supported the hypothesis 1 and 2. 

**Figure 1:**
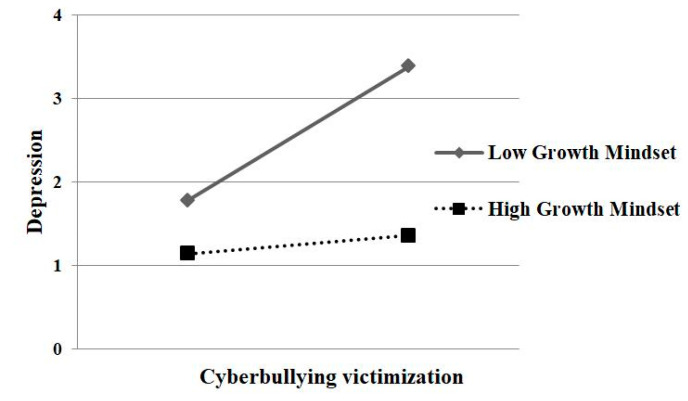
Growth mindset moderated the relationship between cyberbullying victimization and depression.

**Figure 2:**
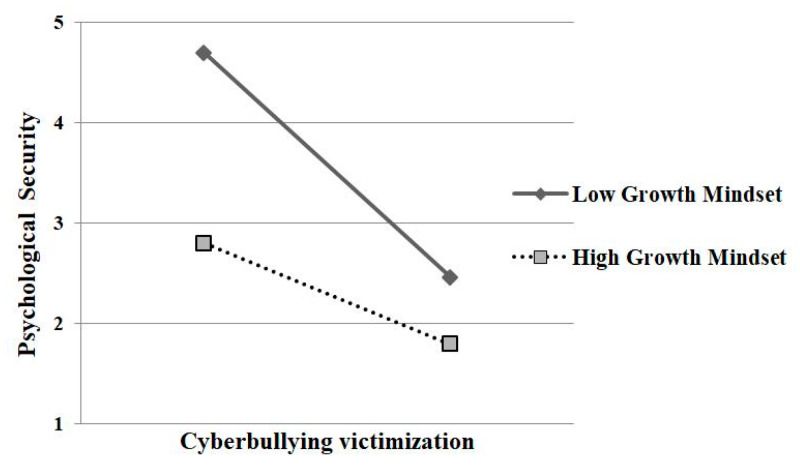
Growth mindset moderated the relationship between cyberbullying victimization and psychological security.

In addition, as can be seen in Table 2, both the interaction effects of cyberbullying victimization and growth mindset on psychological security (*β* = −0.22, *p* < 0.001) and on depression (*β* = −0.18, *p* < 0.001) were significant, indicating that growth mindset moderated the association between cyberbullying victimization and psychological security as well as the association between cyberbullying victimization and depression. At the same time, the results of the conditional direct effect analysis and conditional indirect effect analysis (based on the values of the moderator: *M* − 1*SD*, M, and *M* + 1*SD*) are presented in Table 3, which indicate that two of the three conditional direct effects and indirect effects (based on the moderator values at both the mean and −1 standard deviation) were significantly different from zero (namely significant). In addition, to further analyze the essence of the moderating effect, a simple slope test was adopted. The results showed that for adolescents with low growth mindset (one *SD* below the mean), cyberbullying victimization was significantly associated with psychological security (*β* = −0.52, *p* < 0.001) and depression (*β* = 0.38, *p* < 0.01). However, for adolescents with high growth mindset (one *SD* above the mean), the relationship between cyberbullying victimization and psychological security was weak (*β* = −0.18, *p* < 0.05), and the relationship between cyberbullying victimization and depression was non-significant (*β* = 0.10, *p* = 0.21) (see Figure 1 and Figure 2). To sum up, the significant direct association between cyberbullying victimization and depression, as well as the indirect effect of psychological security in this relationship, was revealed among adolescents with average or low levels of growth mindset but was observed to be non-significant among adolescents with high levels of growth mindset, which indicates that as the level of growth mindset decreases, both of these effects become stronger. These findings supported the hypothesis 3.

## 4. Discussion

Based on the real life of adolescents and the findings of relevant studies, the present study examined the association between cyberbullying victimization and depression, as well as psychological security and growth mindset as two potential mechanisms underlying this relationship. This study, by shedding light on both how and when cyberbullying victimization is associated with depression, could advance our understanding of the detrimental influences of cyberbullying victimization. Moreover, focusing on the underlying mechanisms, this study may also provide a broader perspective for developing prevention and intervention programs regarding depression among adolescents.

Similar to previous studies on the deleterious influences of cyberbullying victimization on individuals [2,3,5,17], this study further indicated that, similar with the research findings on bullying victimization in real life, cyberbullying victimization is also a risk factor for depression. It is well-established that good social relationships (such as peer acceptance and high-quality friendships) can act as protective factors against depression, while negative social relationships (such as bullying victimization and social exclusion) can increase the risk of suffering from depression [12,14,44,45]. Based on previous studies, this result also suggests that the functions and influences of online social interactions and relationships are similar to those of real-life social interactions and relationships [46,47]. Furthermore, this finding also supports the view that being bullied online is a negative and stressful experience, which could cause similar harmful effects like traditional bullying in real life, and that more attention should be paid to cyberbullying victimization among adolescents and its influences.

Furthermore, this study also uncovered how cyberbullying victimization is linked to adolescent depression by discovering that psychological security is a critical mediating factor. The important role of psychological security in terms of individual adaptation has been widely examined [22,23]. According to the cognitive vulnerability model of depression, maladaptive cognition and beliefs regarding self and the world (e.g., low self-esteem and self-worth) are key susceptibility factors contributing to depression [48]. Individuals with poor psychological security usually hold these negative and maladaptive cognitions—perceiving the outer world and other people as threatening, untrustworthy, and uncontrollable [24,25,26]. A lack or a low level of psychological security is closely associated with depression; thus, adolescents with lower psychological security are more likely to suffer from depression. At the same time, adolescents usually develop their sense of security from positive relationships and interactions with others [21,30], while negative interpersonal experiences (such as being victimized or excluded by others) can lead to negative feelings, as well as negative perceptions and beliefs about interpersonal relationships. For example, they may feel scared and helpless, have low levels of social efficacy, and possess little control over social interaction (especially interpersonal adversity) [17,49]. Relevant studies also found that bullying victimization damages psychological security [11,28]. Due to its unique features (e.g., the anonymity of the bullies) that are different from those of traditional bullying, cyberbullying victimization is more uncontrollable and stressful [3,4]. Studies also found that cyberbullying victimization was a great antecedent factor for various negative feelings and cognitions (e.g., anxiety and hopelessness) [2,17,29]. Therefore, cyberbullying victimization results in a decrease in psychological security (namely a feeling that they are isolated or unsafe and that interpersonal adversity is uncontrollable) and further leads to higher levels of depression. This finding also fits well with the main points of emotional security theory [30]. Namely, negative interpersonal contexts threaten individual psychological security, which in turn leads to maladaptive outcomes. The results expanded this theory by focusing on cyberbullying victimization. 

It should be noted that adolescents are usually bullied by their peers, either online or in real life [3,5,14]. However, the role of peer relationships is particularly predominant in adolescence, and adolescents are extremely sensitive to negative social experiences both online and offline [9,50]. Thus, cyberbullying victimization, as one of the repeated, chronic, and uncontrollable stressors, has more serious influences on individual psychological security and depression. 

Nevertheless, we also found individual differences in the associations by examining the buffering or moderating effect of growth mindset. This is one intriguing aspect of the findings: both the direct association between cyberbullying victimization and depression and the indirect association through the mediating effect of psychological security were moderated by growth mindset. In particular, growth mindset could alleviate the adverse effects of cyberbullying victimization on psychological security and depression. This finding is in line with the main points of the diathesis–stress model of depression [6,31] and the stress-buffering model [28,39]. Both of these theories emphasize the important role of the interaction between a stressful context and personal traits in individual adjustment, since it was these interactions that determined the outcomes of the negative experiences. Individuals with positive traits (e.g., growth mindset) may be more adaptive when facing negative experiences. A possible explanation for this is that growth mindset is an important positive personal trait, which refers to the belief or notion that personal attributes (e.g., personality, strength, and skill) are improvable rather than immutable [33,36]. As a basic point of view for understanding and interpreting our attributes’ behaviors [32], mindsets usually determine individual responses to setbacks and adversities to some extent. For individuals with high growth mindset, they believe that their abilities and other attributes can be improved through their own efforts, so when facing negative experiences, they not only demonstrate more resilience and grit, but also show a positive attitude and response. For example, they might seek the necessary social support or take direct action to deal with their problems [36,38], which could help them to effectively cope with negative events and experiences like being bullied online. Additionally, a growth mindset also affects one’s attributions to negative events. Adolescents with high levels of growth mindset will not adopt maladaptive attributions (e.g., self-blaming attributions), but usually have a positive self-attitude [37,51] that can protect them from being too involved in the negative effects of stressors. Thus, adolescents with high growth mindset tend to objectively consider the victimization experiences after being bullied online and take more positive and active countermeasures to deal with the bullying and the painful feelings caused by it, instead of harshly blaming themselves or exaggerating the sufferings. In other words, growth mindset could significantly buffer against the negative influence of cyberbullying victimization on depression and psychological security. This finding is also in line with previous studies indicating that growth mindset is a positive moderator in the association between negative stressors and psychological discomfort [37,38]. The current study, however, extends these previous findings by focusing on a specific online negative experience (i.e., cyberbullying victimization) instead of academic failure and negative events in adolescents’ real daily lives.

### Limitations of the Study

It is important that we should acknowledge some limitations of this research. First, though based on theoretical and empirical evidence, causal inference cannot be made due to the nature of the cross-sectional design. In future studies, experimental, prospective, or longitudinal methods are needed to identify the causality. Second, future research should further consider other individual traits rather than just growth mindset. Recent research has also suggested that the connections between mindsets and mental health are somewhat domain-specific [35,37], and therefore, more targeted methods of measurement are also needed. Third, the effects of social relationships on psychological security may be different in different cultures [52]. In future research, diverse participants from different cultural backgrounds are required to examine potential cultural differences. Finally, this study only focused on cyberbullying victimization. Traditional bullying and victimization should also be included at the same time because they are also common in real life, and there is a close relationship between traditional bullying and cyberbullying [5,14]. Future research should control for traditional bullying or examine the effects of the interaction between traditional bullying and cyberbullying victimization on individuals’ mental health.

## 5. Conclusions

To sum up, the present study enriches our understanding of the mechanisms linking cyberbullying victimization to depression among adolescents: psychological security is a critical mediator linking cyberbullying victimization to adolescent depression, and growth mindset is a positive individual difference factor moderating this association. Growth mindset could especially alleviate the direct influence of cyberbullying victimization on depression and the indirect effect through the mediating effect of psychological security. The mediating role of psychological security and the moderating role of growth mindset together contribute to uncovering the answer to how cyberbullying victimization is associated with depression and when this association is more pronounced or weaker. At the same time, these findings also expand the role of growth mindset as one of the protective factors to buffer against the deleterious influences of online social experiences.

In addition, these findings provide empirical evidence for formulating programs aimed at reducing the negative influences of cyberbullying victimization, as well as for developing the prevention and intervention programs of depression among adolescents. First, developing procedures to improve growth mindset in adolescents could be effective in alleviating the detrimental effects of cyberbullying victimization (i.e., depression and low psychological security), since studies found that growth mindset could be enhanced through offline and online programs [48,53]. Second, considering the direct and mediating effects of psychological security, more attention should be paid to adolescents lacking security in terms of interventions for depression and other outcomes induced by cyberbullying victimization. For example, we might help these individuals by guiding them to view negative social experiences in a more objective and positive way and encourage them to find or seek social support from potential resources to repair and compensate for their sense of security. Last but not least, we should also be aware of the seriousness of cyberbullying victimization and take comprehensive measures to reduce the incidence of cyberbullying.

## Figures and Tables

**Table 1:** Descriptive statistics and correlations among the main variables.

Variables	*M*	*SD*	1	2	3	4
1. Cyberbullying victimization	1.25	0.31	1			
2. Psychological security	3.52	0.61	−0.39 ***	1		
3. Growth mindset	3.15	0.66	−0.22 ***	0.30 ***	1	
4. Depression	1.64	0.49	0.36 ***	−0.48 ***	−0.29 **	1

Notes: ** *p* < 0.01, *** *p* < 0.001.

**Table 2:** Regression models analysis.

	*R* ^2^	*F*	*β*	BootstrapLLCI	BootstrapULCI	*t*
*Model 1 (Outcome: PS)*	0.23	30.35 ***				
Gender			0.08	−0.01	0.18	1.91
Age			−0.05	−0.11	0.03	−0.98
CV			−0.26	−0.33	−0.16	−6.73 ***
GM			0.19	0.10	0.27	4.23 ***
CV × GM			−0.22	−0.32	−0.13	−5.96 ***
*Model 2 (Outcome: Depression)*	0.39	49.52 ***				
Gender			0.10	0.03	0.15	2.31 *
Age			0.06	−0.02	0.10	1.55
CV			0.17	0.08	0.28	3.97 ***
PS			−0.36	−0.49	−0.25	−8.92 ***
GM			−0.15	−0.22	−0.003	−2.47 **
CV × GM			−0.18	−0.30	−0.09	−4.02 ***

Notes: Gender: 0 = male, 1 = female; PS = psychological security, CV = cyberbullying victimization, GM = growth mindset; Bootstrap sample size = 5000; LL = lower limit, CI = confidence interval, UL = upper limit; * *p* < 0.05, ** *p* < 0.01, *** *p* < 0.001; all the variables in the analysis were standardized.

**Table 3:** Conditional direct and indirect effects analysis.

GM Values	Value		BootstrapSE	BootstrapLLCI	BootstrapULCI
*M − SD*	Direct effect	0.39	0.04	0.30	0.47
	Indirect effect	0.20	0.02	0.12	0.27
*M*	Direct effect	0.19	0.03	0.14	0.23
	Indirect effect	0.12	0.02	0.05	0.21
*M + SD*	Direct effect	0.09	0.02	−0.01	0.15
	Indirect effect	0.05	0.02	−0.02	0.09

Notes: GM = growth mindset; Bootstrap sample size = 5000; LL = lower limit, CI = confidence interval, UL = upper limit.
